# New Strategies to Improve the Quality of Life for Normal Aging versus Pathological Aging

**DOI:** 10.3390/jcm11144207

**Published:** 2022-07-20

**Authors:** Manuela Violeta Bacanoiu, Mircea Danoiu

**Affiliations:** 1 Department of Physical Therapy and Sports Medicine, University of Craiova, 200207 Craiova, Romania; mirceadanoiu22@gmail.com; 2 Department of Laboratory Medicine, County Clinical Emergency Hospital of Craiova, 200642 Craiova, Romania

**Keywords:** physical activity, tools, health and well-being lifestyle older adults, neurodegenerative pathology aging, tele-rehabilitation, health nutrition, quality of life

## Abstract

In the context of the manifestation of the phenomenon of normal aging and functional decline at older adults with neurodegenerative pathology, the development of physical activities and healthy lifestyle has become a priority that involves many decisions and responsibilities. Therefore, the study of the quality of life of the elderly in terms of delaying early aging and improving the lifestyle of patients with neurodegenerative diseases is a scientific challenge representing research of great interest and relevance. By promoting physical activity based on telerehabilitation programs or performed according to coordinated training either in the community or at home for both study groups, significant improvements have been obtained. The aim of this paper was to evaluate the intervention suitable patterns, surveys delivered through variables online platforms and tools to reflect the stagnation of early aging and the evolution of patients with PD and dementia. Our study involved selected original studies, intensively processed, which demonstrated through intervention specific tools, such as quantitative, qualitative, socio-economics, physical, and cognitive indicators, that significant improvements can be achieved in the process of early aging, but also significant progress in patients with neurodegenerative diseases. By searching the last five years of papers, our review, presents the importance of intervention by telerehabilitation or by scheduled physical exercises quantified by specific indicators.

## 1. Introduction

Well-being and quality of life represent a multidimensional concept that incorporates aspects related to the functional capacity and mental health of individuals. In terms of older people, the definition of quality of life is similar for other age groups, but physical and mental abilities are much more vulnerable in older adults. However, past studies reveal that the normal (healthy) ageing is not a disease, but is often associated with functional and cognitive decline. The last decade of research was focused to identify the proper interventions aimed to maintain or achieve, as long as possible, the physiological/healthy ageing. On the other hand, ageing is associated with an increased risk of comorbidities, such as neurodegenerative diseases. Pathological ageing appears in aged people affected by an age-related disease. For these persons, the QoL must be oriented to recover or prevent the loss of function progression and maintain or increase the self-independence and social interactions. Nowadays, it is well known that depression is a frequent complication of older adults affected by age-related diseases and social isolation due to the loss of motor functions and/or increased risk of falls can aggravate the outcome and increase mortality.

Functional capacity was defined as the possibility to carry out properly current and autonomous activities both at home and in the environment [1]. Thus, justifying the concern for the evaluation of the physical and mental capacities of this segment of the population. Therefore, new strategies will need to be developed by specialists to prevent premature aging and reduce the decline associated with the elderly or people with neurodegenerative disabilities (pathological ageing). Through these concerns, it is possible to reduce the risk of falls that can create impairments as well as diminish the potential for institutionalization [2]. The risk of falling in the elderly population is worrying and it happens several times a year and, therefore, the concern for its prevention must be continuous. Therefore, new strategies or programs will have to be applied to prevent falls. Their consequences can be accompanied by significant disabilities that require long periods of hospitalization and then costly recovery treatment. Falls similarly affect the elderly population by race or ethnicity. For example, in the USA, almost every hour, three adults die from falls, and the forecast is that by 2030 this will increase to seven [3]. For example, the growing Hispanic/Latino population in the U.S. is growing, and it is imperative to focus on developing programs that prevent of falls. However, first you need to make a program to assess the needs in these communities, to estimate the necessary costs but also to assess the physical abilities of this segment of the population [4]. The risk of falling into normal aging is the biggest public health problem for this age group, being associated with the request of the emergency departments, prolonged hospitalizations or even death. Falling disabilities are a financial burden on health systems that are having to use large sums for hospitalizations and eventual recovery. Thus, the most effective interventions will have to be identified through non-governmental organizations or specialized primary care services to create cost-effective and financially applicable programs to meet the needs of healthy elderly adults in order to increase their well-being and quality of life [5]. Although prolongation of healthy life remains an important public health goal, it is very important to maintain functional and cognitive performance with emotional significance and the ability to live independently during the late life. Insufficient assets are associated with an increased risk of major non-communicable diseases and all-cause mortality, which is linked to increased healthcare costs. For example, people over the age of 65 claim 30–40% of their total health care expenses in Europe. The promotion of physical activity programs organized with variable intensities and constantly applied will succeed in fulfilling their objectives [6]. For older adults with neurodegenerative disabilities such as Parkinson’s disease or Alzheimer’s disease, the major concern is to be able to act as effectively as possible in stopping the evolution of diseases. This is possible through various ways of rehabilitation processes aimed to improve motor and cognitive functions ensuring a quality of life as close as possible. Interventions with assistive devices, both at home and remotely, and of effective protocols for rehabilitation of balance, posture control or improvement of motor functions are aimed to improve the functional outcome. Moreover, motor or non-motor learning programs will, therefore, prevent functional and cognitive decline and increase performance skills of daily activities. Parkinson’s disease is associated with impairment of gait parameters, decreasing skills ability, instability postural, and risk of falls. Injuries from falling create the fear of moving and the time diminishes the motor capacities determining the accentuation of the disabilities. At the same time, the accentuation of the functional decline is accompanied by the cognitive one; therefore, the efforts to maintain the two functions cooperatively is even greater. Regarding mild cognitive impairments, organized programs for monitoring the physical activities evaluated with different tools will have to be applied, in order to prevent the cognitive decline and to increase the abilities in performing ADL and to increase the life expectancy and its quality. In this sense, for the neurodegenerative diseases of the elderly, the public health problems, and the costs related to their rehabilitation will be even higher. Interventions scheduled for physical and mental activity, at home, in primary care institutions with specialists in the field or by telerehabilitation will have to be a priority of public health systems with interventions from governments or other non-governmental associations around the world. Constantly sustained physical activity with exercises for balance, endurance, and strength, by applying programs delivered on online platforms, such as Tai Chi or Nordic walking, allows improvement of motor and cognitive functions and reduces the risk of falling, which remains an acute problem for this segment of elderly patients [7].

## 2. Materials and Methods

### 2.1. Search Criteria

In this study, we searched the most relevant publications in Pub Med and Cochrane databases, using keywords such as “physical activity”, “tools”, “health and well-being lifestyle older adults”, “neurodegenerative pathology aging”, “telerehabilitation”, “health nutrition”, and “quality of life”. Our search was performed using key words combination as follow: (older adult) AND (health nutrition) AND (wellbeing life style); (older adult) AND (health nutrition) OR (wellbeing life style) OR (telerehabilitation) AND (neurodegenerative diseases); (neurodegenerative diseases) AND (ageing) AND (cognitive function rehabilitation) AND (wellbeing)**.** The selected clinical trials, pilot study, observational and prospective studies, meta-analyses or reviews, and original articles or other papers was performed in accordance with the Prisma flow diagram [8]. The most representative full-text studies published in the last five years were considered. In all clinical trials and original articles, the intervention of physical activity was followed both through organized programs developed at home based, in communities or in centers with specialized assistance and delivered through online platforms both for healthy elderly and senior patients with motor and cognitive impairments from neurodegenerative diseases. Furthermore, we included in the final selection, the relevant articles selected from the reference list Only English language publications were considered.

### 2.2. Selection Strategy

Several guidelines have been used for this qualitative synthesis. (1) Individuals from our study were healthy older adults and elderly with motor and cognitive impairments from Parkinson’s disease (PD) and Alzheimer’s disease (AD). (2) The tools for assessing well-being and quality of life were grouped as follows: quantitative general tools, qualitative specific tools, socio-economics tools, physical tools, falls tools, and cognitive tools. All of these instruments have been applied to both normal aging and pathology aging; (3) all randomized studies used in the synthesis included a large number of individuals of both sexes; (4) physical interventions were performed both at home, or under specialized assistance in different centers and delivered through online platforms or smartphones in the form of semi-structured interviews or through dedicated programs that using different devices; (5) physical trainings which using exercises with different intensity, were applied for both groups by older adults; (6) in this study, both experimental and control group were included; (7) interventions through telemedicine and telerehabilitation for normal aging and normal pathology have identified the need for interventions through new technological strategies that can prevent the decline of the functional abilities of seemingly healthy elderly people and can delay the progress of seniors with PD and AD; and (8) ways to assess real mobility of elderly people in everyday life and access to various facilities outside the home. Exclusion criteria for these papers were: other comorbidities which may occur in these age groups, various surgeries, and older adults who needed permanent assistance.

### 2.3. Dynamics of Extracting Significant Data

From the electronic databases, 340 relevant writings from Pub Med and 69 papers from Cochrane, have been selected from the last 5 years, which address the delay of the morphological, functional, or mental decline of healthy elderly people but also the improvement of motor and mental health deficiencies in the older adults with PD and AD. The aim of this research is to increase the expectation of well-being and quality of life and to reduce the need for care for both groups of the elderly. After the elimination of duplicate papers, 405 studies remained to be scanned. Subsequently, another 194 records were removed that represented descriptions of older adults with comorbidities other than motor and non-motor disabilities in PD and AD. A total of 211 full-text clinical trials and original articles were ultimately considered eligible for qualitative synthesis. Of these, 177 studies were excluded for the following reasons: 110 papers were systematic reviews, 6 writings were pilot study, 2 were referring to a case study, 4 were abstract studies, 3 were drafted in other languages, 7 were observational and prospective studies, 7 papers were not free to access, 15 did not approach physical activity, and 23 studies did not research elderly problems. Finally, in qualitative synthesis, 34 clinical trials and articles, which addressed healthy older adults and telerehabilitation for seniors with neurodegenerative diseases, remained eligible for study.

### 2.4. Model Quality Synthesis Assessment

Several variables were used regarding selection and extraction of data: (1) reference and data publication; (2) the intervention variables tools for assessment primary and secondary outcomes; (3) the presence assistive devices both live and telerehabilitation; (3) features of the physical activity were performed at home-based, in specialized health care centers or specialized healthcare centers or remote through the technology of online platforms to present appropriate patterns or eHealth interventions through online questionnaires and semi-structured interviews; (4) design of interventions regarding the protocols used for physical training or against falls; and (5) methods used, results or discussions, and conclusions from each writing.

## 3. Results

**Figure 1:**
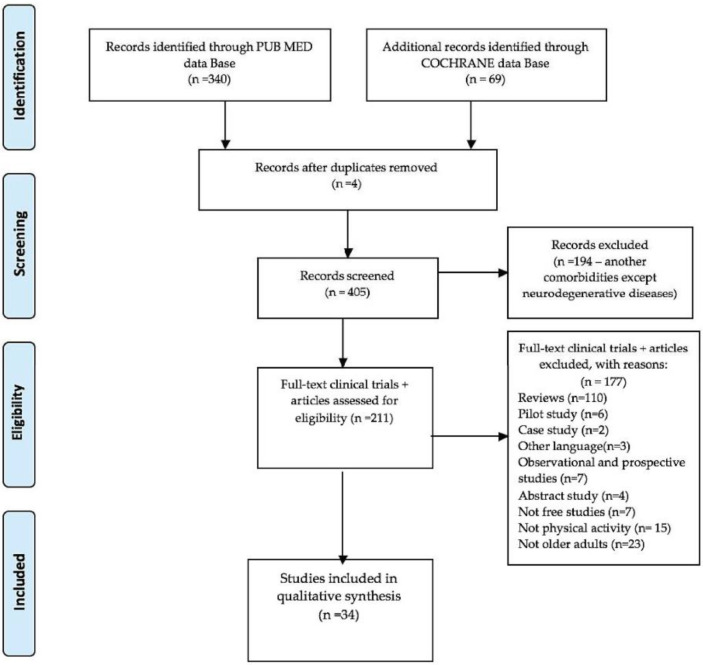
PRISMA 2009 flow diagram.

The dynamic presentation of qualitative research is highlighted in Figure 1 of the PRISMA 2009 flow diagram:

### 3.1. Tools/Instruments—Determinants of Well-Being and Quality of Life in Seemingly Healthy Elderly and Older Adults with Neurodegenerative Diseases

The quality and well-being life of the elderly is influenced by various factors, of a social and economic nature, but also by factors that reflect individual and biological characteristics or life history so that four categories of factors are identified: (i) health, (ii) socio-economic circumstances, and (iii) psycho-social circumstances and demographic characteristics. Regarding the health of older adults, long-term limitations, difficulties in carrying out daily activities, depression, sleep disorders or reduced functional capacity manage to strongly influence the well-being and quality of life of these people. Socio-economic factors that may have a positive effect on delaying the decline of well-being or ameliorating impairments in seniors with PD and AD are correlated with access to durable goods, ownership or education that may have significant effects on quality of life. The negative effect is reflected in the life expectancy advanced by the low level of income. Psycho-social circumstances with beneficial effects on the quality of life of the elderly are associated with the dimension of social interactions, with the consolidation of interpersonal relationships and the intense affective participation of family, children, or friends. All these determinants in a favorable sense contribute to the composition and maintenance of a well-being of physical and mental health necessary in the knowledge and monitoring of the quality of life of the elderly. Thus, health and good motor and mental functioning, the availability of personal utility, participation in social events, cooperation in interfamilial and intergenerational relationships as well as social and economic support provided by appropriate services are decisive indicators of quality of life in the elderly. Investigating the impact of physical and mental health on the quality of life of older adults will need to be conducted on the caregivers of the elderly, highlighting the differences between those living in the community and those in residential care institutions. The environment can stimulate or impair the emotional state of the elderly with considerable effects on mobility, sensory sensitivity or cognitive impairment. That is why the living environment is a very important factor that can stimulate the independence of the elderly, the consolidation of personal identity or the increase of self-confidence. Thus, the tools needed to assess the quality of life can be classified as follows: (1) quantitative general tools/instruments; (2) qualitative specific tools/instruments; (3) socio-economic tools/instruments; (4) physical tools/instruments; (5) falls tools/instruments; and (6) cognitive tools/instruments. All of these indicators are used for both seemingly healthy seniors and older adults with neurodegenerative diseases (Table 1).

#### 3.1.1. Quantitative General Tools/Instruments

The instrument which investigated five or three dimensions, such as mobility, self-care, daily activities, pain/discomfort or anxiety/depression, registered on a visual vertical analogic scale, is EuroQol-5D-3L. The results can be used as a measure quantitative state of health that reflects perception the person. The scores for the five dimensions can be presented in the form of a health profile or can be transformed into an index, according to methodologies provided by researchers who have contributed to the creation of the instrument [9]. AQol-6D/NeuroQol, QUALY, HRQol, Mini-OQLO, SWB, are instruments which quality of life related with health status. Dimensions refer to independent living, happiness, mental health, opportunities for managing difficulties, pain, self-worth, and emotions [10,11,12,13,14,15,16]. For each dimension there are 3 severity levels, which are: “no problems”, “some problems” and “extreme problems”. The person is asked to indicate their state of health by checking the box next to the level most appropriate to the state of health corresponding to each of the 5 dimensions. The vertical analog visual scale records the patient’s health on a visual scale, where the points endings are labeled “best health possible” and “the worst possible health condition” [12,15,17,18]. SF-36 is a tool which measures quality of life related with health conditions. The categories covered are physical health, vitality, social relationships, emotional troubles, mental health or impairments psychological, or well-being life [15,19,20]. Other instruments, such as PSS, PROMIS, HAAS, FSF, and Neuro Qol, generate information about physical activity, proper nutrition and stress, and mental health [11,21,22]. GLFS with 25 items evaluated body pain, difficulty with movement, regular care, social activities, cognitive status, and daily activities [23]. For PTSD and RPE, mental health was investigated in older adults which included stress disorders post traumatic [24]. Other tools that assess the health and disability of seniors are WHODAS, COMPAS-W, and SNAO [13,25]. The most important tools for quantifying dietary benchmarks are DRV, MUFA, PUFA, RIR, FI, and RI, which investigated the possibility of using a proper diet with a good balance between energy needs and daily consumption, as well as food intake recommendations based on metabolic needs induced and physical activity [26]. The size of the scales for healthy eating are extremely important: m SOF, a HEI, MED, DASH, and FFQ, which these tools refer to, and these tools refer to the implementation of appropriate diets verified by questionnaires that quantify the energy consumed daily and which are recommended for the prevention of hypertension [27].

#### 3.1.2. Qualitative Specific Tools/Instruments

The instrument ICECAP-O/ICEpop, was used for assessing the quality of life from an economic perspective. The tools are correlated with the well-being of life given by the individual’s ability to be or do things that are important in life. The questionnaire includes questions about affection, friendship, security, satisfaction, or independence [18,28,29]. Moreover, in this sense, other instruments, such as SUS, MSEQ, LQS, LSS, SRSE, or MRSE have been defined, correlated with the satisfaction of the quality of life with certainty and a good mobility of evolution [11,14,18,30]. RAVLT is a neuropsychological tool used to assess functions, such as memory, attention, and learning ability in the auditory-verbal domain [25,31]. Other tools, such as HHIE, IOI-AI, BRCS, and PEG evaluated different impairments and investigated issues of general activities [11,19]. Quality of life for patients with Parkinson’s disease was monitored through PDQ-8 [32,33]. Well-being and status health was assessed through PHQ-4 [24,32]. The COSI tool is used to estimate the qualitative improvement of the elderly person’s life [19].

#### 3.1.3. Socio-Economics Tools/Instruments

Effective interventions to increase the quality of life and well-being of older adults are appreciated through the following tools: CEA, CCA, CCWBA, ICER, VIF, and COPM. Therefore, the development of various support programs for the delay of early aging, as well as the promotion of rehabilitation activities for the elderly with neurodegenerative diseases, will reduce maintenance costs and will make recovery for aging pathology more efficient [12,15,29,34,35]. For example, Canadian public health care systems are constantly concerned with reducing maintenance costs and increasing the effectiveness of senior rehabilitation by promoting the most sustained physical training at home, in order to reduce the pressure on public health services systems [15].

#### 3.1.4. Physical Tools/Instruments

The motor performance for healthy older adults and seniors with neurodegenerative diseases can be evaluated using focused tools. In order to investigate motor abilities and gait performance, the following scales were used: SPPB, and TUG, which investigated motors ability beginning for 3 m walking then turn and back walk and stand of chair [4,10,13,15,18,22,25,31,35]. For assessing parameters of gait used instruments, such as 5TSTST, 6 min walk test, 4,10,400 m WT, SEW-D, DGI, TLS, MDRT, MSL, and scales that investigate walking at the highest speeds, track the length and frequency of the step and their completion times [10,15,19,25,31,33,36,37]. Quantifying balance and posture both statically and dynamically is extremely important in assessing the motor and functional status of the healthy elderly adult, but also of the older adults with neurodegenerative impairments. The most appreciated tools in this regard are BBS, scale with score range from 0–56, which assess balance tasks [14,20,33,38]. The scale BOOMER measures outcomes in elderly rehabilitation [10], ABC, BARSE, SEE, SIBT, COP, and One foot balance test, give confidence in balancing activities [19,22,33,37,39] and other tools, such as PASE, Grip strength, Back scratch, and ROM, appreciate the varying degrees of physical activity performed by older adults [14,19,22,25,31]. Training dual task was appreciated through DT-FPT, SOT, MDC, and FRT [22,33,40]. The instruments which measure progression diseases are: UPDRS, Hoehn and Yahr, and ALF-FRS-R. Fatigue induced by physical activities of various degrees of intensity is evaluated by instruments such as VAS, PFS [14,18,41].

#### 3.1.5. Falls Tools/Instruments

In the case of elderly people prone to reduced functional autonomy, the risk of falling becomes more pronounced. The negative impact of these events on the quality of life is well known, and, therefore, the need to improve the tools needed to quantify falling trends in healthy elderly and older people with neurodegenerative impairments. For instance, BADL and IADL represent indicators related with falls [16,39,42]. Fall risk scores are investigated by FRAT, FaB, FRM, and PROFANE [4,14,43]. Fear of falling was evaluated through FES-I and Short FES [10,13,14,28,31,32,40,41]. Different physical activities may be accompanied by risks of falling after several stages of training that can be assessed by: FSST and Mini BEST 28 [36].

#### 3.1.6. Cognitive Tools/Instruments

Aging can be accompanied by both the appearance of motor dysfunctions and the alteration of cognitive behaviors. The available tools used to monitor the non-motor or cognitive disorders were focused on different directions. For example, MMSE assesses cognitive functions impairments [25,30,38,41,43]. Mental disorders will intervene in the evaluation DSMV and MoCA [22,30]. The evaluation of the psychological profile is performed by PPA and FCSRT [13,22,28,30]. Other tools will investigate the varying degrees of depression, such as GDS, PHQ-9, and CES-D [10,11,13,19,22,25,28,43]. PDSS-2 assesses by 15 items on a visual scale the quality of sleep in older adults with PD [32] and the degrees of mental disorder are assessed by CRIq and D-QOL [22,30,38]. Monitoring the impairment of cognitive function with aging through these tools justifies the concern regarding the quality-of-life conditions in the elderly either healthy or with neurodegenerative diseases.

### 3.2. The Impact of Recovery Strategies through Physical Activity and the Use of Assisted Devices on the Well-Being and Quality of Life for Normal Aging

The complexity and multidimensionality of the concept of quality of life in the elderly is identified by the main determinants of well-being, namely: age, psychological or emotional issues, health status that refers especially to functional autonomy and mobility, participation and social support, and a sense of security, but also the living environment. The dimensions of the quality of life are different for dependent elderly people who benefit from social services with or without accommodation in home care units or in day care and recovery centers, compared to seniors who can lead an independent life.

**Figure 2:**
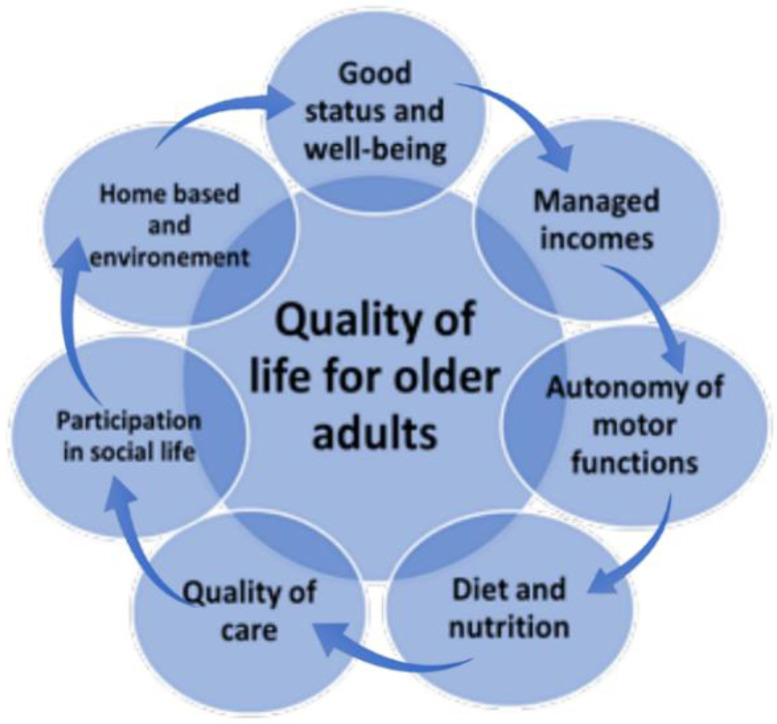
Multimodal approach of Quality of Life in older adults.

Promoting sustained physical therapy in various programs, as well as duration and frequency of the physical exercises, both for the dependent elderly population and for those with independent living, is one of the most important strategies to prevent premature aging and sustain a functional autonomy and an appreciable quality of life. Recommended exercise programs for older adults can be combined with assisted devices that are highly recommended for gaining motor benefits for both safe walking and the development of muscle strength, intensity of movement and postural safety and dynamic balance. The physical activity was very varied, starting from daily domestic activities and reaching physical exercises based on recommended programs for walking training, static and dynamic balance but also physical therapy to improve muscle strength for both upper and lower limbs. Physical training has been recommended to be performed daily, several times a week, and with a certain frequency and intensity to obtain benefits (Figure 2).

We present below Table 2 which is relevant for those discussed above.

Two papers, the authors who used the Sitless study, discussed walking training, performed over a period of 16 weeks, 5 times a week, using recommended exercise schemes for the elderly with sedentary behavior. The physical effort was of a mixed aerobic and anaerobic type, starting from physical exercises to maintain balance and a steady gait to the stimulation of muscular strength by using the elastic band dynamometer, bike ergometer or actigraph accelerometer. Socio-economic tools, such a: ICECAP-O, ICEpop, CEA, CCA, and CCWBA, that monitor physical activity, have demonstrated an effective economic model that has reduced additional costs and assessed health status for older adults by highlighting its improvement through the recommended physical program, administered with adequate frequency and intensity [18,29]. Implementation of the program PACE-IT (physical and cognitive exercises intervention) and DT-FPT (dual task functional power workout) demonstrated a decreasing trend in falls and improving balance and gait, and enhancing power and velocity of contraction for hands muscles. Weights were used in the hand or transported over different distances in exercises administered during a 26-week period, thus stimulating two different tasks through corroborated physical activities [40]. In our study, two articles are analyzed in which the participants are exclusively elderly women with postmenopausal osteoporosis. The promoted physical activity starts from a program of physical exercises with intensities that increase progressively and are performed over a long period of time. The physical exercises performed refer to improving the balance, to sitting and lifting from the chair, to walking with increasing distances and intensities progressively, or to evolutions regarding the strength or mobility of the joints. Various tools used in evaluating the results have been demonstrated improving of pattern gait, coordination balance and motricity, joint mobility, and enhancing performance of motion upper limbs [10,11]. One paper discusses elderly people with hearing impairment with representation for both sexes who use hearing aids. It is very important to intervene in a coordinated physical program with exercises to strengthen the muscles to increase endurance and physical endurance. The program walk, talk, listen, which is the pattern of audiological rehabilitation (GAR), was improved communication strategies with stimulation health education [19]. Other authors who discussed diet used older adults. The articles referred to two population groups in either urban or rural areas who eat healthy or have a poor diet. In both cases, there is the benefit of aerobic physical activity or ADL’s to prevent the tendency for age-specific falls, or certain scheduled physical exercises that support motor and cognitive functional autonomy for older adults. The need for the future is to implement programs to promote health and healthy diet for the elderly in both urban and rural areas, regardless of their income and, especially, to combat poor socio-economic status. [7,10,12]. In another study, the authors promote the rationalization of the use of physical activities as efficiently, as possible by approaching a physical program, Tai Ji Quan where interventions are such as: the best mobilizations for a safe balance, multimodal exercises to stimulate safe walking and functional autonomy, but also those of endurance that can strengthen the muscles on the belts of the upper and lower limbs of the elderly population [8]. Another program, called Pisando Fuerte, developed in Spanish, promotes physical activity with exercises for strength and safe balance and prevents risk of falls at older population living independently. In this regard, it was promoted as a session learning workshop lasting two hours in eight weeks, and assistive devices used were weights for legs who ensure a good exercise and prevent the risk of falling [9]. In order to have good physical and mental health, it is necessary to maintain a body weight appropriate to age and physiological needs. That is why bold intervention programs have been developed through physical activities capable of causing weight loss in older adults where appropriate. Aerobic exercise training who used by recumbent stepper, stationary bike and treadmill, reused for 12 weeks with 30 min/session, to ensure an adequate weight necessary for the functional autonomy and well-being of the elderly adult. By means of tools such as PHQ-9, DSMV, PTSI or RPE, physical and mental health was monitored for sedentary seniors [11]. In another study, the authors investigated, through Locomotive syndrome risk test, walking exercises while maintaining stance using different weights: 10, 20, 30, 40 cm, with major benefits in improving dysfunction on lower limbs [23].

### 3.3. The Impact of Telerehabilitation through Physical Activity with the Use of Assistive Devices for Apparently Healthy Adults and Seniors with Neurodegenerative Diseases

In this section, we aim to summarize from the analysis of available studies, the modalities of intervention by telerehabilitation, using technological models with applications in this regard, for apparently healthy elderly and elderly adults who have neurodegenerative impairments. Therefore, we will discuss, separately, the two directions of telerehabilitation through assistive devices, both for the apparently healthy elderly population who have been or have not been hospitalized or who are institutionalized and for elderly patients with neurodegenerative diseases that can be supported at home. Our goal is to identify the most effective strategies for sustaining well-being and quality of life and preventing premature aging for older adults, as well as to improve the dysfunction of elderly patients with neurodegenerative disorders and to promote mental and physical health in this regard.

#### 3.3.1. Telerehabilitation Sustained by Assisted Devices for Efficient Physical Activities at Normal Aging

Continuing exercise programs at home through technology platforms after discharge from the hospital, have been shown to be very effective in improving strength and motor skills in the upper and lower limbs, but also in preventing falls. Thus, through physical therapy performed for balance and endurance with 3–4 sessions/week, home-based, and delivered through a smartphone application, the balance of walking performance was improved, as well as the increase of resistance and strength at the level of the limbs. The physical activity included squats, kneeling, floor pushup, forward, and lateral lunge, maintaining balance on single leg [1]. Through the “Back to my Best” protocol, delivered via video or telephone application, including multimodal exercises that prevent falls, elderly adults continued at home for 3 months after hospitalization with daily educational and physical activities for 15–30 min. The beneficial results showed a decrease in fall rate by about 30% [5]. The program PACE (protocol of all inclusive care of elderly) applied at home showed improved walking speed and balance safety. The instruments that proved these advantages were SPPB, TUG, and FCI [4]. The Standing Tall, promoted by the My Compas technology platform with health education intervention on tablets in improving motor and cognitive performance, decreasing the risk of falling, and increasing the well-being and quality of life, proved to be just as effective. The physical activities carried out over 52 weeks focused on cognitive motor training balance [2,9]. The concern for the prevention of premature aging and quality of life was aimed in addition to telerehabilitation through monitored physical programs and the application of an adequate diet delivered through educational programs applied through self-care or virtual protocol applied by nutrition instructors. In one of the articles, we discuss with physical training carried out in 12 weeks with 136 min/week, about self-care plan regarding diet and sleep. The protocol aimed at weight loss of participants without additional effort, but with the performance of planned physical exercises, performed regularly and with a healthy and balanced diet [10]. On the other hand, another author monitored 184 older adults through the CALM program, which aimed to improve balance and gait performance, as well as the use of an adequate protein, vitamin intake in order to combat the decrease in muscle mass [7]. In another study, the authors were concerned about the well-being and quality of life of elderly physicians who were instructed by a virtual program assisted by therapists and nutritionists to perform proper physical activity or dieting for which the daily caloric intake was calculated [3]. The concern for improving the quality of life from older housing or sheltered housing aimed to prevent functional and cognitive decline. The risk of falling in these individuals being high required the development of educational learning protocols and the promotion of effective physical activities using tools for recording movements in the environment. The walking parameters were, thus, improved, managing to increase the speed of movement and the width of the step which promoted a safe balance and prevented falls [6]. In another study, as a fall prevention strategy, the beneficial intervention of devices that provide exergames that can stimulate the safe balance and the improvement of strength and physical endurance, through exercises performed at home carefully monitored. The assessment was performed using scales that monitored the risk of falling or fear of falling and assessed the balance of the elderly, such as BBS, FRAT, ProFANE, or Short FES. The use of platforms that deliver exergames has proven to be efficient and financially easy to use, and extremely educational [8]. Available tools for assistive devices are presented in Table 3.

#### 3.3.2. Telerehabilitation through Physical Therapy Sustained by Assisted Devices at Older Adults with Neurodegenerative Disorders

Neurodegenerative diseases are accompanied by varying of deficits that affect motor activities, and spatial-temporal parameters of gait, balance, postural stability, and cognitive functions. Considerable efforts have been made in recent years to promote and use non-pharmacological therapies, such as complementary or alternative therapies, that have been shown to be effective in improving daily activities, gait analysis, postural instability or reducing the risk of fall, and, finally, enhancing quality of life and well-being. The randomized controlled studies, from last five years, have discussed Parkinson’s disease (PD), Mild cognitive impairments (MCI) or Vascular cognitive impairment (VCI) by Alzheimer’s disease. Along with the benefits through physical therapies with the participation of active devices, telerehabilitation is an alternative variant that through exergaming or video games combines body movements with the multiple game skills in virtual realities. Participation in balance training mediated by Nintendo Tele Wii consisted of exercises that improved postural reactions, voluntary or anticipatory movement strategies even if the training is quasi-static and is mainly based on self-stabilization tasks. The comparison is made between the benefits achieved by promoting rehabilitation through virtual reality at home with physical therapy for the decline of postural instability using sensory integration balance training (SIBT). Home-based virtual reality (VR) balance training proves to be an effective alternative remote therapy for postural improvements, enhancing cognitive functions, amelioration of balance disorders, and improved gait intervention in PD (2,5–3 for H&Y) with caregiver assistance. Through VR, positive results for BBS and DGI, related with group SIBT were observed [22]. Older adults with PD usually suffer from postural instability, leading to poor balance and an increasing risk of falls. Exercise-based video games training (exergaming) are a form of physical treatment that is delivered through virtual reality technology to facilitate motor learning and is effective in improving balance in elderly populations. The exergaming is used to provide static and dynamic balancing exercises for people with PD, using Jintronix Software with virtual rehabilitation from a personal computer or laptop. In this randomized study via the Jintronix app, a transcranial stimulation was applied in the experimental group in two sessions per week for 12 weeks. Each VR training session used exercises for static and dynamic balance for 20 min The results were evaluated at 6 weeks during the intervention, at 12 weeks after the intervention, and at 3 months after the end of the stimulation. The primary outcomes generated results through limits of stability test, and the secondary ones included measurements of static balance, strength of legs, and motor functional capacities, which demonstrated improving cognitive tasks. The secondary outcomes were shown through using m BEST that assesses four domains of dynamic balance anticipatory postural adjustments, reactive control postural, sensory orientation, and dynamic gait. The improvement of the scores in the other instruments, such as 5TSTST, 10 MWT, and 4SST, demonstrated the effectiveness of the intervention through a-t DCS (anodal transcranial direct current stimulation), which indicated enhancing motor learning, and real benefits in static and dynamic balance, as well as reducing falls [33]. In the other randomized pilot study, the beneficial intervention of electroacupuncture was evaluated using LEG Sys (wearable sensor technologies) SEIRIN- stainless steel acupuncture needles (EA) and ITO-ES (electric stimulators) on gait analysis. The physical exercises were performed for 30 min, 3 weeks in once weekly. The primary outcomes were evaluated gait speed and secondary results were showed the positive interventions in gait parameters, such as stride length, cadence, double support, and swing angular velocity. The benefits were demonstrated through FES-I, VAS, and MMSE, with obvious improvement in scores. The results proved to be spectacular in the analysis of gait in terms of STHW (single task habitual walking) and DTFW (double task habitual walking), STFW (single task fast walking), and DTHW (dual task fast walking). In conclusion, EA treatment had multiple benefits especially with dual task walking [36]. Telerehabilitation has been shown to be equally effective through mHealth application, which connected in another randomized controlled study with 148 participants with early or mild PD, who underwent a physical program aimed at improved strength muscle of motor skills related to the number of steps performed per day, as at the time when more than 100 steps are performed per days. Step Watch TM 4 Activity Monitor (SAM) worn 24 h of day, excepting showering or bathing, registered daily steps counts and number of minutes in which the participant more than 100 steps in seven consecutive days. Physical training was applied for 30–60 min, for one session in 12 months, which was of comprised lunge and squat sets, heel raise or step-ups, and exercises for multidirectional stepping [41]. Primary outcomes will include the change between baseline and 12 months in overall amount of walking activity (mean number of minutes per day) and amount of moderate activity walking training (mean number of minutes per day in which >100 steps were accumulated). Secondary outcomes will include change in walking capacity as measured by the 6 min walking test and 10 m walking test (6 MKT, 10 MKT) where walking for 6 or 10 m at the highest speed was evaluated. The assessment was made through BARSE, which is a self-administered questionnaire and assesses the participant’s confidence in walking. Is a very important in this study, because intervention of the graded physical effort which appreciated in terms of increasing the number of steps performed per day, but also the walking intensity which considerably improves the walking skills and its strength [41].

In a quasi-randomized prospective longitudinal study, it was demonstrated that tablet performed training program app, ruled at home, applied for 9 months after regular schedule MKT improved motor skills, reduced non-motor impairments, and enhanced quality of life. Although MKT is a real activity training (Nordic walking, dancing, or Tai Chi) together with physiotherapist intervention, it was observed that tablet program app, which comprises video images with explanations and instructions for all physical exercises by MKT, addresses very well for strength training, balance, mobility assessment, and endurance. Primary outcomes evaluated quality of life at PD older adults through PDQ-8, which contains eight items related to daily activities, about comfort, mental state, communication support, and various motor activities. Secondary outcomes evaluated all restrictions due to impairments from chronic illness through IMET, fear of falling through FES-I or sleep disorders (PDSS-2), and depression, anxiety, and emotions by PHQ-4 [30]. In another cross-over trail, which was used in intervention group a technique video games home-based exercises, at mild or moderate PD, through Xavi X entertainment app, who has been shown to be effective in improvement postural stability, there were positive results in dynamic balance and reducing incidence of PD falls. The influence balance training program through IVGB (interactive video game based) was ruled at 6 weeks and evaluation was made after 12 weeks after physical activity. Evaluation of primary BBS and secondary outcomes such as MFES, which quantified falls trend; MDRT, which appreciates the strength to arrive in left, right, and forward directions; MSL, which represents an indicator mobility and falls functions risk; SF-36, a real self -reported questionnaire which evaluates physical, emotional, social functions, general vitality, and mental health, demonstrated improvements in postural stability, balance or reducing incidence of falls. IVGB is a program exercise consisting of two tasks: a multi-directional step task and a step-by-step task aimed at the target. The system of IVGB provides audible and visual feedback in both tasks, which increases participant’s attention. First, the participant followed the illustrated instructions displayed on the monitor to trend the target area to complete the multidirectional step load. The first task evaluates the participants’ ability to change weight, dynamic balance, and stability. The second task evaluates the participants in terms of movement coordination and balance while older adult he stands on one leg. Adjustments were made between three levels of difficulty and the direction of the steps by a specialist according to the participant’s cognition, attention, balance, gait, strength, and ability to change weight. To ensure uniformity in the exercise posture, older adults were asked to maintain an upright position and to avoid rocking compensation [20].

In the MCI or VCI by Alzheimer’s disease, the intervention of telerehabilitation has been shown to be just as beneficial as other neurodegenerative diseases. The home rehabilitation program was performed in 8 weeks, including cognitive for 3 days of week, APA (Adapted physical activities) for 2 days/week, and social actions for once a week. Evaluation of cognitive functions is performed through Serious Games (SGs) developed by Brian HQ app, which belongs to the platform Posit Science using an actigraphy (GENEActiv), which monitors ADL, quality of sleep, and GOAL app architecture. The primary outcomes, such as MoCA, the digit span and Corsi span tasks, FCSRT, IFR, RCFT, MCST, TMT, semantic, and phonetic fluencies, showed changes in cognitive functions, which addresses the cognitive domain and secondary outcomes were appreciated the changes of physical and social actions through CESD. The cognitive module means assessment of attention and executive functions, visuospatial ability, semantic and phonetic fluency, intelligence, memory, neural plasticity, and skills ability. The physical module was comprised of physical activity delivered through APA exercises. The physical activity means aerobic and anaerobic training for upper and lower limbs, for trunk, neck, stretching sessions, force exercises, muscles strengthening, and resistance treadmill delivered through video games. The social module was represented by daily activities, such as gardening, cooking, showering, watching a movie, and other weekend activities, transmitted through questionnaires which need completed after each activity [22]. Another randomized controlled trial investigating Alzheimer’s disease by physical activity over 6 weeks, 8 weeks, and a follow up 12 months, for 5 days/week with tablet based cognitive activity and 7 days/week for aerobic exercises. Telerehabilitation was conducted using the following tools: a tablet which had items for cognitive activities, including language, memory, attention, visual and spatial skills, executive functions and other instruments; a sphygmomanometer for the detection blood pression, pulse oximeter which measures saturation oxygen, heart rate, scale for body weight; and a Fit Bit which quantified load physical and sleep activity. Through the ability program developed on the tablet app, beneficial effects were highlighted for mental and physical activity at older adults with cognitive impairments [37]. Through RedCap software app., promoted an educational material regarding shoulder health in ALS, with beneficial results for improving motricity and strength developed in upper limbs, as well as relief of ALS pain in the shoulders. The assessment of the evolution in terms of improving the motor activity of the upper limbs and reducing pain at this level is done by estimating ROM as a primary outcome or by questioning using 5-point Likert scale as a secondary outcome [44].

Below, we will find synthetized the telerehabilitation with assistive devices at neurodegenerative pathology for older adults in Table 4.

## 4. Discussion

Quality of life is a multi-level and complex concept that includes aspects associated with functionality and physical, mental, emotional, social, and personal perception. Life satisfaction and well-being are more than the actual health of the body and lack of disease, and are a sum of aspects related to physical health, mental health, emotional health, financial situation, occupation, education, social life, and environment. The concept is related to satisfaction life, successful ageing, subjective well-being, and happiness. In the age of personalized medicine, multidisciplinary therapeutic approach strategies are required that can be individualized. Therapeutic management addresses the key factors that can affect the outcome of therapy, and which are often personalized. For the success of recovery therapy, the degrees of comorbidity must be appreciated, demographic factors that can limit compliance and access to therapies as digitalized as possible. In this sense, it is necessary to use adequate multidisciplinary rehabilitation programs applied especially in stages of medium or moderate disease such as Parkinson’s disease. In general, multidisciplinary interventions are effective in both the functional and cognitive fields. The improvement of daily activities being correlated with the severity of motor dysfunctions is relevant by improving stiffness and bradykinesia. In particular, by improving motor functions in the upper limbs, it will increase manual dexterity in performing and maintaining daily activities, improving verbal comprehension, visuo-constructional abilities, or creating new skills. Beneficial effects have also been observed in global cognitive functions, through multiple integrated interventions: physical, speech or occupational therapy. All these will delay the progress of the general neurodegenerative process. at the individual level the improvement of cognitive performances such as visual and verbal memory, visuo-perceptual and visuo-constructional skills and language were evident. The multiple needs of patients with neurodegenerative diseases are multifaceted; therefore, the approaches of multidisciplinary rehabilitation management must be individualized considering the stage of the disease. Unfortunately, in advanced disease stages, the improvement of functional and motor results is irrelevant. Therefore, the intervention of multidisciplinary rehabilitation will have to be considered in early or moderate PD stages, in order to delay the progression of motor and non-motor disabilities, and hospitalizations and rehospitalizations will increase the financial costs [45,46].

Mono- or multidisciplinary programs to support normal aging or those of conventional rehabilitation face to face for aging pathology are addressed through the nursing services of a limited number of participants. Therefore, the need for new strategies to promote quality of life and well-being for healthy elderly adults or for functional and cognitive rehabilitation for aging pathology has proven to be fundamental. Thus, the programmed digital technology was promoted and subsequently developed, which managed, through different platforms, the delivery of standardized protocols or digital rehabilitation programs that substantially contributed to the improvement of the quality of life or the improvement of neurodegenerative symptoms. The real advantage of these digital technologies applied to physiological or pathological aging is that they apply to a very large number of participants. On the other hand, the support of digitally delivered health programs, as well as rehabilitation protocols developed at home based and monitored remotely by therapists, required a medium or even high educational degree in order to be implemented. In another light, promoting and implementing digital strategies to support health for older adults or rehabilitation for pathology aging can be more financially efficient thus avoiding overloading healthcare services. Telerehabilitation, tele-monitoring, and tele-engagement have proven to be effective digital solutions for promoting the quality of life of well-being and physiological aging, but also beneficial strategies for functional and cognitive rehabilitation pathology ageing. Telerehabilitation sessions through double-loop communication, applied with a certain frequency and intensity developed through engaging digital platforms with endurance training or dance therapy have allowed beneficial results in ameliorating motor or cognitive dysfunctions in patients with neurodegenerative processes [47].

The aim our study was to monitor the intervention of physical activity with assisted devices both applied life or through telerehabilitation, to increase the well-being and quality of life for seemingly healthy elderly adults, and for the elderly with neurodegenerative disorders. The two directions have been shown to be very effective in delaying premature aging, as well as in ameliorating neurodegenerative dysfunctions, with effective benefits for motor and cognitive functions in the two types of older adults studied. In both cases, there were appropriate tools for assessing primary and secondary outcomes, as well as assisted devices assigned directly or at home, which demonstrated significant improvements in motor and cognitive skills for the apparently healthy elderly and those with neurodegenerative pathology. It is noteworthy that almost similar assessment tools were used for both strategies, and the physical activities served by specialized protocols applied directly or through technological platforms, generated almost similar beneficial effects for functional and cognitive autonomy.

For instance, in two articles, the positive intervention for SITLESS study, regarding combating sedentary lifestyle, and promoting a healthy lifestyle, with the intervention of the live physical activity protocol with recommended exercise schemes that maintain the physical condition and well-being of the body were discussed. At the same time, an economic model of long-term health promotion for the elderly has been developed, which will combat sedentary lifestyles, and will implement learning programs for physical and cognitive rehabilitation, both for the institutionalized and for those assisted at home [18,29]. For protocol Locomotive Syndrome risk test, which was a support for care of the elderly, the exercises training, consisted of two steps while maintaining balance at different weights, monitored by GLFS with 25 items for questionnaire; the advantages were for the delay of motor functions deficiencies and ability skills, especially for the lower extremity [23]. There are even the same programs used for both live and telerehabilitation, such as PACE-IT, which investigates dual task functional power training, but which addresses different assistive devices. Although different physical activities are approached, the two directions have a common goal, that of improving balance, gait parameters, and decreasing the incidence of falls, but also skills in strength and speed of movement [35,40]. A new approach to motor and cognitive stimulation is achieved through the intervention of gaming technology. Assistive devices such as OTAGO Fame games, Tele Wii Nintendo protocol, XaviX entertainment system with IVGB, and Jintronix Software, have succeeded both through direct use or delivered through the online platform to bring important benefits in improving balance, muscle strength, decreasing the incidence of falls, and stimulating neuroplasticity, but also profitability and efficiency in terms of the costs necessary for these strategies [14,20,33,36]. An important strategic direction to discuss is the issue of diet for the older adults. Delayed premature aging and the progression of neurodegenerative disorders are associated with an adequate protein diet that prevents the decrease of muscle mass, low in fat and carbohydrates that reduce weight gain, but also with an adequate vitamin intake. There are even programs developed in this regard such as Calm, Change, Physicians health study, or Self-care weekly, that intervene effectively in these directions. Associated with a balanced nutrition you will have to intervene with a scheduled physical activity capable of stimulating the intensity of the aerobic response, increasing strength and speed of motor skills, and weight loss where it is required for a healthy lifestyle. In this sense, health policies will have to be approached to promote a functional diet in both rural and urban areas, but also financial support for those who benefit from low incomes, who cannot provide a healthy diet [21,25,26,27,34,42]. Another problem associated with senescence is bone fragility, especially in postmenopausal women, which can be a common cause of spontaneous fractures or frequent falls. Two authors discussed the intervention of physical programs of different intensities, carried out regularly and for extended periods of time, such as Taiji with the help of devices such as dumbbells and stack weight devices, which managed to delay the installation of motor suffering, pathological fractures, and improving balance coordination with motor activity [10,11]. However, most authors were concerned with investigating the incidence of falls, their risk, and their prevention for healthy adults with neurodegenerative impairments. Programs such as “Standing Tall” or “Back to my Best” intervened by telerehabilitation with devices that delivered cognitive behavioral or motor training, that improved quality of life through decreasing rate of falls. At the same time, there has been an increase in physical activity that has promoted balance, motor skills and even cooperative functions [13,16,28]. Exercises performed for balance and strength were applied by the Pisando Fuerte protocol and by Tai Ji Quan multimodal exercises and stretching program are used. The results were favorable regarding decreasing tendency of falls and amelioration the parameters of gait. Another advantage was the reduction of therapeutic costs by reducing the risk of falling, but also the delay of aging by stimulating mobility and cognitive functions [4,12]. Monitoring physical activity with a motion sensor installed at home and using a digital dynamometer demonstrated the delay of motor and functional decline and the decrease in the tendency to fall [43]. Another study investigated cognitive reserve index by applying questionnaires after performing the rehabilitation protocol with breathing education, stretching activity, mobilization of the joints of the upper and lower limbs that showed improved gait and balance, and reduced risk of falling [38].

## 5. Conclusions

Older adults have the right to be at the center of the process of determining well-being and quality of life. However, the elderly did not ensure a homogeneous group, due to the intervention of pathological situations; in recent years special attention has been paid to research investigating the impact of functional and mental autonomy on the quality of life of older adults. In this sense, guidelines have been developed for the promotion of physical activity in all its forms, which achieve beneficial motor and cognitive effects, both for apparently healthy adults and those with neurodegenerative deficiencies. In terms of classical physical activity, it will have to be continued and perfected through well-composed physical programs, at a higher pace, but with a shorter time. Assistive devices intervention will consistently improve gait parameters, static and dynamic balance, range of motion, postural reflexes and muscle strength and endurance. The quality of life and well-being will be determined by a good mental function sustained in the same sense with coordinated physical activities for the two types of older adults.

Home based telerehabilitation should be extended using interactive games video, on tablets or using other applications, such as Kinect Sensor Otago, My Compas, Vita Kost, Madlog, Eat More, Brian HQ app, Goal app architecture, GaitRite Mat digital, actigraphy, dumbbells, bicycle ergometer, treadmill, RAVLT, grip dynamometer, accelerometer, and weights, thus stimulating daily activities improving in motor skills and cognitive functions.

It is very important to obtain a decrease in weight loss and slow progression of diseases through the interventions of telemedicine using hypercaloric feeding applications.

The training through video exercises proved to be attractive, delighted the older adults, and managed to improvement both the physical and mental status.

One of the biggest achievements was the decrease of the falling trend and the state of fear induced by it.

Telerehabilitation offers possibilities for stagnation of physical, cognitive, and social decline for healthy older adults or seniors with neurodegenerative disorders, through applications developed that are easy to handle and with the active participation of caregivers and specialists.

However, there are two very sensitive bridges that should be mentioned: older adults living in disadvantaged areas, dwelling communities, discharge hospitalization, or housing care, for whom travel to specialists would be difficult due to financial expensive; and there are limitations on the costs of instruments/tools used or the extend periods of time allocated to effective monitoring of remote older adults by physicians and care givers. To these is added the incorrect assessment of the needs of the elderly and the degree of dependency, which makes the interventions only formal.

Telerehabilitation is mainly addressed to a segment of older adults who are intellectually trained and able to master new technologies because these applications require material and mental skills.

Our study reveals a new perspective on the e-Health platform, which will need to be further expanded to be able to inform older adults about social care services, long-term home care strategies.

For this support to be possible, the public authorities responsible for this field will have to be involved, as well as non-governmental organizations capable of financially stimulating projects necessary to prevent premature aging or to improve neurodegenerative disorders in older adults.

## Figures and Tables

**Table 1:** Well-being and Quality of life Tools/Instruments.

Quantitative General Tools	Qualitative Specific Tools	Socio-Economics Tools	Physical Tools	Falls Tools	Cognitive Tools
EuroQoL-5D-3L HRQoL AQoL-6D/NeuroQoL SWB QUALY Mini-OQLQ SF-36 FCI PSS FAP GLFS PTSD RPE WHODAS SNAQ DRV MUFA PUFA RIR RI FI mSOF aHEI MED DASH FFQ PROMIS COMPAS-W	ICEpop/ICECAP-O SUS LQS LSS SRSE MRSE RAVLT MSEQ HHIE IOI-AI BRCS PEG PDQ-8 HPQ-4 COSI	CEA CCA CCWBA ICER VIF COPM	SPPB TUG Chair sit to stand Test/5TSTST 6 min Walk test One foot balance test Grip strength Back scratch BBS PASE VAS Fatigue 4, 10, 400 m WT BOOMER TLS PFS DT-FPT ABC SOT FRT TMT MCST ROM IPAQ SIBT MDC DGI BARSE SEW-D MDTR MSL UPDRS H&Y ALF-FRS-R COP	BADL IADL FRAT ShortFES FES-I PROFANE Fab FRM FSST Mini Best-28 SEE	Jong Loneliness MMSE GDS DSMV PHQ-9/4 PPA MoCA FCSRT CES-D CRIq PDSS-2 D-QoL

**Quantitative general tools**: EuroQol -5D-3L: including five dimensions: self-care, mobility, usual activities, pain/discomfort, anxiety/depression; HRQoL: Health related with quality of life; AQoL-6D/NeuroQoL: Assessment quality of life, with dimensions: independently life, mental health, happiness, relationships, self-efficacy, pain, difficulty management; SWB: Subjective wellbeing, which measures quality of life related to health conditions; QUALY: Quality adjusted life years; Mini-OQLQ: mini osteoporosis quality of life; SF-36: Physical functioning Scale, measures physical health, pain, vitality, social relationships, mental disorders; FCI: Functional comorbidity index; PSS: Perceived Stress Scale; RPE: rating of perceived exertion; PTSD: Scale-post-traumatic stress disorder; FAP: Functional Abulation profile; GLFS: Geriatric locomotive function scale; WHODAS: WHO disability assessment schedule related dietary intake (protein, fiber, fruits, vitamins); SNAQ: Short nutritional assessment questionnaire; DRV: Dietary reference value; MUFA: mono-unsaturated fatty acids; PUFA: poly-unsaturated fatty acids; RIR: Recommended intake range; RI: Reference intake; FI: Fraitly index; mSOF: Frailty score-modified study of osteoporotic fractures; aHEI: Alternative healthy eating index; MED: Mediterranean diet; DASH: Diet approaches to stop Hypertension; FFQ: Food frequency questionnaire (energy intake kcal/day); PROMIS: Patient reported outcomes measurement information system; HAAS: Harvard Alumni Activity Survey. **Qualitative specific tools:** ICEpop/ICECAP-O: Capability measure for older: evaluating the quality of life from an economic point of view; SUS: System usability Scale life; LQS: Life quality Scale; LSS: Life satisfaction Scale; SRSE- Self regulatory self-efficacy; MRSE: Mobility regulatory self-efficacy; MSEQ: Marcu’s self-efficacy; RAVLT: Rey auditory verbal learning Test; HHIE: Hearing handicap inventory for the elderly; IOI-AI: International outcomes inventory alternative intervention; BRCS: Brief resilience copy Scale; PEG: Pain, Enjoyment and General Activity; PDQ-8: Parkinson’s Disease Quality of Life questionnaire; PHQ-4: Health Questionnaire for Patients. **Socio-economics tools**: COSI: Client oriented Scale of improvement; CEA: Cost effectiveness analysis; CCA: Cost consequence analysis; CCWBA: Cost capability wellbeing analysis; ICER: Incremental cost effectiveness; VIF: Variance inflation factors; COPM: Canadian occupational performance measure: socio-economical outcome. **Physical tools**: SPPB: Short Physical performance battery; TUG: Time Up and Go; Chair sit to stand Test; 6 min. Walk Test; One foot balance Test; Grip strength; Back scratch; BBS: Berg Balance Test; 4, 10, 400 m WT: 4, 10, 400 m Walk test; PASE: Physical activity Scale for Elderly; VAS Fatigue: Visual Analog Scale of fatigue; BOOMER: Balance outcome measure for Elder rehabilitation; TLS: time loaded standing; PFS: Pittsburgh Fatigability Scale; DT-FPT: Dual -task functional power training; ABC: Activity balance confidence scale; SOT: Sensory organization test-evaluating postural ability through visual, somatosensory and vestibular system; FRT: Functional reach Test < 18 s evaluating limitation skills ability for upper limbs; TMT: Trail Making Test; MCST: Modified Card Sorting Test; ROM: Range of motion; IPAQ: International Physical Activity Questionnaire; SIBT: Sensory Integration Balance Training; MDC: Minimal detectable Change; DGI: Dynamic Gait Index; BARSE: Barriers Self-Efficacy Scale; SEW-D: Self-Efficacy of Walking Duration; MDRT: Multidirectional Reach Test; MSL: Maxim Step Length; UPDRS: UPDRS: Unified Parkinson’s Disease Rating Scale; H&Y: Hoehn & Yahr; ALS-FRS-R: Amyotrophic Lateral Sclerosis Functional Rating, revised; COP: Centre of pressure. **Falls tools**: BADL: Barthel Index correlated with falls; IADL: Lawton Index correlated with falls; FRAT: Fall risk score; Short FES: Falls Efficacy scale; FES-I: Short form falls efficacy Scale- fear of falling; PROFANE: monitoring falls; FaB: Falls behavioral risk scale; FRM: Fall risk measure; FSST: Four square Test evaluating risk of falls at 15 s; Mini- BEST 28: Mini balance evaluation system test 5 × SST -five times sit to stand test of falls (>15 s); SEE: Self -efficacy for exercise Scale. **Cognitive tools:** MMSE: Mini mental State Examination; GDS: Geriatric Depressive Scale; DSMV: Diagnostic and statistical of mental disorders; PHQ-9: Scale for evaluation depression; PPA: Physiological profile assessment; MoCA—Montreal Cognitive Assessment; FCSRT: Free and Cued Selective Reminding Test; CES-D: Centre of Epidemiological Studies Depression Scale; CRIq: Cognitive Reserve Index Questionnaire; PDSS-2: Parkinson’s Disease Sleep Scale; D-QoL: Dementia Quality Life Instrument.

**Table 2:** Positive effects of measured outcomes in physical activity with assistive devices in healthy older adults.

References	Design	Physical Activity	Primary Outcomes	Secondary Outcomes	Assistive Devices	Conclusions
Deida M., et al. (2018) [29]	SITLESS study 1338 participants > 65 years	30 min/daily times 5/week walking training	ERS-exercise referral scheme, SB-sedentary behavior PA-promoting physical activity	EQ-5D-5L ICECAP-O, ICEpop CEA CCA CCWBA	Actigraphy, Dumbbells Bike ergometer Elastic bands Grip dynamometer	Developing health economic model of older adults on long term, Evaluation healthcare and news orientation for promoting healthy programs with a rate of decreasing expenditures.
Duckham R.L., et al. (2018) [40]	PACE-IT 398 participants > 65 years 110-male 288-female	26 weeks exercise training 45–60 min/session: motion exercises, balance training, HV-PRT	Falls rate Risk rate	Changes in lower limb motricity, Grip strength, Dynamic balance, Quality of life, Gait, Cognitive functions Short FES	Hand grip dynamometer, Handheld weights, Tubing, Sands bags, Thera-bands, Weighted vests	Improving balance, gait pattern, Decreasing trend falls, Enhancing strength and velocity motions, Increasing skills in knee motility, dorsiflexion and grip strength.
Gibbs J.C., et al. (2019) [10]	Osteoporosis Number of falls in past year >75 years 71-intervention group female 70-control group female	Physical exercises training from to moderate and high intensity 6–16 min/week	SPPB 4 m Walk Test 5-TSTS-5 TUG TLS BOOMER	EQ-5D-3L Mini OQLQ FES-I	Dumbbells	Developing the new strategies for performing physical activities which improve strength, joint mobility and the possibility of delaying the onset of osteoporosis in postmenopausal, Improving performance gait and upper limbs motricity, Early assessment and prevention of bone aging as well as postural outcomes at women elderly.
Giné-Garriga, M., et al. (2017) [18]	SITLESS study 1338 participants > 65 years	16 weeks walking training 45–60 min/session 32 sessions times two/week	ERS—exercise referral scheme, SB—sedentary behavior PA—promoting physical activity	SPPB Muscle functions Health economic factors QUALYs EQ-5D-5L ICECAP-O Anthroponomy items Cognitive functions MSEQ Questionnaire Fear of falling Fear of falling Physical fatigue PFS	Hip work Actigraphy accelerometer Weights Grip dynamometer	Promoting strategies to combat sedentary behavior for the elderly population by implementing exercise schemes and supporting exciting learning programs capable rehabilitating physical and cognitive functions, Decreasing in the costs of institutionalized or assisted home care for the elderly.
Jones C.A., et al. (2019) [19]	Hearing loss 35-intervention group GAR+ 31-control group GAR >65 years 57%—male 43%—female	10 weeks—SHE strengthening exercises 60 min strength, resistance, walking and coordination training	Functional outcomes: Chair sit to stand Test 6 min Walk test TUG One foot balance test Grip strength Back scratch	Psycho-social outcomes: HHIE SF-36 GDS IOI-AI COSI	Hearing aids Pedometer	WTL (walk, talk, listen) Improving communication strategies, Health education, Major benefits through fitness exercises for integration in society, GAR represents the first confidence the decreasing loneliness, Improving functional (gait speed, strength, resistance, health education) and emotional conditions, Increasing social contacts, Improving emotional and social loneliness.
Klein D., et al. (2017) [34]	CHANGE Canadian health advanced by nutrition and graded exercises Metabolic syndrome 307 participants 40–76 years	Physical aerobic exercises 3 sessions/week 20–30 min/session	Change to fitness Flexibility Strength	COPM	Treadmill	Improving capacity and mobility of motion, Enhancing intensity of muscular exercises further with a nutrition healthy, Increasing aerobic response and strength of motility, Decreasing risk cardiovascular for older adults.
Li F., et al. (2017) [12]	TJQMBB 218-multimodal exercises 363-stretching exercises >70 years Impairments mobility History of falling	24 weeks twice training/weekly Tai Ji Quan intervention-moving for better balance Multimodal exercises Stretching physical activity	Number of falls	EQ-5D-5L QUALYs ICER	Weights Resistance tubing	Streamlining mobility through TJQMBB intervention, Decreasing the tendency to falls in the elderly and reducing the costs of therapeutic intervention and aging delay through different degrees of physical activity.
Mora Pinzon M., et al. (2019) [4]	PISANDO FUERTE Living independently History of falls 24 participants ≥ 65 years 13%—male 87%—female	Physical exercises for balance and strength Session learning workshop 2 h in 8 weeks	FaB (*p* ˂ 0.0001) TUG (*p* = 0.07)	Fidelity performing in learning program Upgrading exercises (regular exercises) Safe walking behaviors Safer stand-up from sitting position Change footwear	Assistive devices- weights using for physical activity	Decreasing tendency to falls 6 months training, Improving stance and strength of motility, Discussions with physicians for medication, Environmental changes which decreasing falls risk, Maintenance and continuation of physical exercises.
Oliveira A., et al. (2019) [42]	HCS-home care services (Portugal) ADHC-adult day care services Income Hospitalization institutional affiliation falls 95-healthy diet 15-poor diet 65–98 years 22%—male 78%—female	ADL Guided exercises Walking 3 times/week 20 min	BADL (*p* = 0.042)	IADL (*p* = 0.047)	Questionnaires: Social and demographic questions health status information	Increasing quality of life of life through care-givers family, Decreasing decline for elderly participants through emotional support, Preventing risk of falls or falls related disorders, sequels with or without change in joints mobility, gait and other motor functions Delaying decline of elderly through physical activity Adopting strategies which stimulate activities daily for older adults.
Pebole M.M., et al. (2019) [24]	Warrior wellness intervention sedentary 60–76 years 41-male 4-female	12 weeks 30 min/session Aerobic exercise Training (recumbent stepper, treadmill, stationary cycle	Weight loss rate	PHQ 9 BMI DSMV PTSD RPE	Bike Treadmill	Improving physical health, mental health, Promoting strategy health with major impact in older adults, Decreasing of weight.
Shahar S., et al. (2019) [25]	SES-socio-economic status Nutritional status 2237 urban and rural group >60 years	ADL Physical fitness	BMI TUG Chair stand test Chair sit test 2 min step test Back scratch test Grip test	MMSE GDS WHODAS Dietary intake (Proteins, fiber, fruits, vitamins)	RAVLT PASW	The need to promote program to improving of health and nutrition for the elderly, Improving physical program interventions in both urban and rural areas for people with low incomes and residents in rural areas, Promoting the care of the elderly by launching new health policies that provide a functional diet for those with poor SES.
Vilpunaho T., et al. (2019) [11]	Osteoporosis 457-intervention group 457-control group >60 years female	6 months training with weekly exercises gym and Taiji session 6 months slowly Exercises 12 months physical activity with low cost 60 min/session 2 times/week	Falls rate Falls risk	EQ-5D-5L LQS LSS GDS PSS BRCS MMSE QoL SWB	Stack weight device Biweekly SMS Questions phone	Reducing falls and fair of falls through Taiji program, Enhancing coordination balance and level motricity, Increasing cost-effectiveness for rehabilitation intervention, Delaying the installation Of mobility impairments the appearance of pathological fractures as well as The subsequent institutionalization or the use of caregivers at home.
Yamada K., et al. (2018) [23]	Locomotive syndrome risk test 1469 participants 76–88 years 1009-male 460-female	Exercises walking strength two steps while maintaining balance at different weights:10, 20,30, 40, cm.)	Two step score (Ratio of length of two steps and elderly height) Stand-up test score	GLFS	Weights	Early identification of ways to delay the decrease of mobility the lower extremity in the elderly population, Delaying the aggravation of the motor functional deficiencies who already have difficulties in the motor skills of the lower limbs, Delaying the institutionalization of the elderly with progressive motor deficit.

**Table 3:** Telerehabilitation through assistive devices for normal aging.

References	Design	Physical Exercises	Primary Outcomes	Secondary Outcomes	Assistive Devices	Conclusions
Borges P.R.T., et al. (2021) [15]	Public health care Discharged from hospitals	Physical therapy for resistance and balance home based 3–4 high intensity session/week	TUG 30-CST-30	SF-36 VAS EuroQol-5D COPM	Smartphone app.	Effectiveness and cost-effectiveness through training telerehabilitation at discharged older adults, Improving strength and resistance for upper and lower limbs, Enhancing balance, Improving gait pattern and skills of motricity.
	115-participants telerehabilitation group 115-control group					
	>60 years					
Delbaere K., et al. (2021) [13]	Standing Tall 114 -participants intervention group 112-control group >70 years	ADL Physical activity training	Rate of falls at 12 months	At 24 months EQ-5D-5L AQol-6 D PPA TMT Icon FES SPPB WHODAS COMPAS-W	Tablet with health education intervention McRoberts Move Monitor	Preventing falls in older adults through E-health program which improving executive and cognitive functions, Decreasing injuries associated falls.
Djuric Z., et al. (2017) [27]	Physicians’ Health Study 82 participants	ADL Vigorous exercises weekly	Frailty index (FI)	mSOF aHEI MED DASH FFQ	Well-coaches training program Mas-Medical assistants virtually program	Evaluating diet factors and implementation vigorous physical activity Decreasing risk of frailty for physician older adults with motors impairments and cognitive disorders.
	Pre-frail group Frail group >60 years					
Falvey J.R., et al. (2019) [35]	PACE program 525 participants >65 years	ADL	SPPB TUG F-CI VIF	Gait speed (m/s) Balance Strength	Stopwatch	Preventing the decrease of physical abilities in elderly adults with different degrees of impairments.
Hill A.M., et al. (2017) [16]	“Back to My Best” Hospital discharge AMT 390 participants >60 years	ADL 45 min/session times 2 sessions of education in hospital 15–30 min/session at phone for 3 months consecutive after for hospitalization	Rate of falls for the first 6 months	Percent of participants with falls Rate of injuries falls Katz index IADL AQOL-6 D	Phone Pre -made Video for 10 min	Decreasing rate of falls about 30%, with educational program “Back to my Best” after hospital discharge.
Naseri C., et al. (2017) [39]	Semi-structured interviews at phone Hospital discharge AMT 390 participants 195- intervention group 195-control group >60 years	ADL IADL Strength training Balance exercises Walking training	Rate of falls Katz index Lawton index	Falls prevention after 6 months Discharging Likert Scale SEE	Phone	Changing of physical behavior in order to prevent falls, Learning patterns educational which falls prevention.
Rantz M., et al. (2017) [43]	Senior housing Hospitalizations Physicians visit 86-intervention group 85-control group >83 years 23-male 62-female	ADL	SF-12 GDS MMSE ADL-IADL Gait Rite FRM FAP Hand grips	Walking speed in 10 s. Velocity (Gait Rite) Stride length right Stride length left Stride length right and left	Gait Rite Digital Dynamometer Motion system sensor non-wearable	Delaying the functional decline by using the warning system through environmental sensors installed at home, Monitoring behavioral activities tendency of falls and parameters gait, Extension of time for institutionalization of the elderly in nursing houses.
Rønnow, Schacht, S., et al. (2019) [26]	CALM 184 participants 65–81 years 79 -male 78-female	Exercises training 400 m walking	DRV MUFA PUFA 400 WT	AR RIR RI	VITAKOST dietary MADLOG app.	Improving the balance in the consumption of foods that bring adequate energy intake such as protein intake, vitamin D and thiamine intake and the application of a constant physical program to the detriment of the use of mono- and polyunsaturated fatty acids, alcohol consumption to increase well-being and prevention early aging.
Stanmore E.K., et al. (2019) [26]	Sheltered housing Good mental capacity Able to TV watch, gaming, technology, speaking English 56-exergame group 50-control group >55 years or older 22%—male 78%—female	12 weeks 30 min/3 times/week Exercises for strength and balance	BBS FRAT VAS	PASE VAS fatigue HR Qol SUS TAM ProFANE QUALY	Kinect sensor ONTAGO-FaME Exergames app.	Improving balance, strength, Decreasing falls and fear falling, Increasing quality of life, Enhancing cost-effectiveness strategies against falling.
van Schooten K.S., et al. (2021) [28]	Standing Tall eHealth intervention CBT-cognitive behavioral training CMT-cognitive motor training 259-health education group 259-intervention group ≥65 years	52 weeks 2–3 h/week Balance cognitive motor training	Rate of falls in the 12 months/person	EQ-5D-5L COMPAS-W WKODAS Exercise training Psychological well-being Balance ability in motricity Health literacy GDS QoL PPA Icon FES ICE pop	Tablets with MyCompas app., CBT program	Improving quality of life through decreasing rate of falls, Enhancing physical activity which promoting balance safety, motor skills, safe walking, but also improving of cognitive functions, Increasing well-being and tomorrow’s safety.
Ward R.E., et al. (2020) [21]	Self-care weekly program regarding diet, Sleep and physical activity	8–12 weeks 136 min/week Physical training	Neuro QoL FSF PROMIS	Confidence to carry out physical activity PSS	Smart phone planning	Improving mobility and cognitive functions, Losing in weight without efforts, Changing of lifestyle for older adults through tele-assistive intervention and learning self-care program household on diet, behavioral sleeping and exercise training.
	39 participants >51 years or older					

**Table 4:** Telerehabilitation with assistive devices at neurodegenerative pathology for older adults.

References	Design	Physical Exercises	Primary Outcomes	Secondary Outcomes	Assistive Devices	Conclusions
Fabbri L., et al. (2019) [22]	Randomized controlled trial study protocol (AD) experimental group 60 participants	8 weeks 3 days/week cognitive Activity 2 days/week APA (adapted physical activity) 1 day/week social activities	Cognitive domain: MoCA FCSRT IFR TMT Stroop Test	Physical and social domains: ADCS/ADL CES-D SF-36 CRIq SPPB HAAS IPAQ	GENEA actigraph GOAL app. Architecture Brian HQ app.	Decreasing cognitive functions, Increasing skills in ADL.
Gandolfi M., et al. (2017) [33]	Randomized (PD) single blind 38—VR group 38-SIBT group	50 min/session 21 sessions 3 days/week 7 weeks	BBS MDC	ABC 10-MWT DGI MCID PDQ-8 5 points Likert Scale	Tele Wii Nintendo protocol (exergaming) SIBT protocol (self- and external destabilization exercises)	Improving balance, gait and postural control, Decreasing number of falls, Reducing postural instability, Declining Impairments cognitive functions.
Harris D. M., et al. (2018) [36]	Randomized controlled trial (PD) 5-exergaming group 19-control group	2 sessions/week 12 weeks Walking training	Limits of stability test	UPDRS H&Y COP mBEST 5TSTST 10 MWT FST PDQ-39	Jintronix Software with a-t DCS (anodal-transcranial direct current stimulation)	Stimulating neuroplasticity, Improving static balance, Facilitating motor learning, Decreasing risk of falls.
Lei H., et al. (2016) [41]	Randomized pilot study (PD) 10-EA-experimental group 5-sham EA-control group	30 min/session 3 sessions 1 day/week training walking	Gait speed	Spatio-temporal gait parameters UPDRS H&Y FES-I VAS MMSE	LEG Sys SEIRIN-stainless steel acupuncture needles ITO-ES-electric stimulators	Performing gait analysis: improving for STHW and DTHW, enhancing cadence, swinging angular velocity.
Rawson K.S., et al. (2020) [41]	Randomized controlled trial (PD) 140-experimental group 8-control group	30–60 min/ session 6 sessions 12 months Walking training Strengthening exercises	Mean number of steps/day Mean number of minutes/day with >100 steps	6MKT 10MKT BARSE SEW-D	mHealth software Step Watch TM 4 activity monitor (SAM)	Increasing walking ability.
Realdon O., et al. (2016) [30]	Randomized controlled trial (AD) ability-group usual care-group	Physical exercises for 6 weeks and 8 weeks follow up 12 months 5 days/week with tablet cognitive activity 7 days/week aerobic activity	SUS D-QoL	MMSE MoCA FCSRT IFR TMT DCS/ADL	Tablet ACG Sphyngo-manometer Pulse oximeter Heart Rate (HR) Fit Bit	Improving motor and non-motor functions.
Siegert S., et al. (2019) [32]	Quasi-randomized prospective longitudinal study (PD)	9 months Physical training MKT	PDQ-8	IMET FES-I PDSS-2 PHQ-4 Questionnaire Shanga	Tablet performed training program app (MKT)	Improving motor skills, Enhancing cognitive functions.
Yuan R.Y., et al. (2020) [20]	Cross-over trial (PD) intervention group-IVGB control group	12 weeks Balance training	BBS	FES-M MDRT MSL SF-36	XaviX entertainment system with IVGB (inter-active video games-based)	Reducing incidence of falls, Enhancing postural stability, Improving static and dynamic balance.
Piccinini G., et al. (2018) [38]	Idiopathic PD UPDRS (III) H&Y CR: cognitive reserve >64 years 28-male 25-female	32 sessions for 4 months 2 times/week 50 min/session Rehabilitation protocol	BBS MMSE	CR level CRI score	CRIq: cognitive reserve index questionnaire education, working activity, leisure time BIT: Brief intelligence Test	Improving gait, balance and reducing risk of falls which undergo a conventional rehabilitation training against with patients who had higher CR.
Burke K., et al. (2018) [44]	Educational material(brochure) regarding shoulder health in ALS 16 participants	Stretching Exercises Strengthening training Sprinting Position strategies with arm on pillow	ROM	Five-point Likert scale	RedCap software app	Improving motion, Strengthening muscle shoulders, Decreasing pain and dysfunction of upper limbs.
